# The complete chloroplast genome sequence of Korean raspberry *Rubus crataegifolius* (Rosaceae)

**DOI:** 10.1080/23802359.2017.1398621

**Published:** 2017-11-07

**Authors:** Ji Young Yang, Jae-Hong Pak, Seung-Chul Kim

**Affiliations:** a Research Institute for Dok-do and Ulleung-do Island, Kyungpook National University, Daegu, Gyeongsangbuk-do, Republic of Korea;; b Department of Biological Sciences, Sungkyunkwan University, Suwon, Gyeonggi-do, Republic of Korea

**Keywords:** Chloroplast genome, Korean raspberry, *Rubus crataegifolius*, Rosaceae

## Abstract

The first complete chloroplast genome sequences of Korean raspberry, oriental herbal medicinal plant of Korea, were reported in this study. The genome size was 155,714 bp, composed with one pair of inverted repeats (IRs) of 25,781 bp, which were separated by one large single copy (LSC; 85,402 bp) and one small single copy (SSC; 18,750 bp). The genome contained 131 genes, coding for 86 proteins, eight ribosomal RNAs, and 37 transfer RNAs. The overall GC content was 37.1%. Phylogenetic analysis suggests that *R. crataegifolius* is sister to *R. corchorifolius*, which belongs to subgenus *Idaeobatus*.

The genus *Rubus* L. consists of approximately 750 species (Hummer 1996). It has been recognized as one of taxonomically challenging groups due to frequent hybridization, polyploidy, and agamospermy (Weber 1996; Howarth et al. 1997; Alice and Campbell 1999). *Rubi Fructus* is the dried immature fruits of *R*. *coreanus* Miq and *R*. *chingii* Hu and is known to exert several pharmacological effects including antitumor, antioxidant, and anti-inflammatory activities. Owing to the pharmacological importance of *Rubi Fructus*, several studies have been conducted for pharmacological effects and molecular marker development of *R. coreanus* and related species (e.g. Kim and Lee 1991; Lee et al. 2015). The immature fruits of common East Asian raspberry species, *R. crataegifolius* Bunge, are often sold as *Rubi Fructus* in the Korean herbal markets (Namba et al. 1986; Yang et al. 2012). Therefore, authentication based on DNA markers is of the utmost importance. As an effort to develop molecular markers for authentication and highly variable chroroplast markers for phylogenetic and population genetic studies, we sequenced the complete chloroplast genome of *R. crataegifolius*.

Approximately 1 g of fresh leaves of *R*. *crataegioflius* were collected from natural population (Voucher specimen: KNU-Yang161107). Total DNA was isolated using the DNeasy plant Mini Kit (Quiagen, Carlsbad, CA) and sequenced by the Illumina HiSeq 4000 (Illumina Inc., San Diego, CA). A total of 41,509,908 paired-end reads were obtained and assembled *de novo* with Velvet v. 1.2.10 using multiple *k*-mers (Zerbino and Birney 2008). The tRNAs were confirmed using tRNAsacn-SE (Lowe and Eddy 1997). The complete chloroplast genome of *R*. *crataegifolius* (MG189543) is 155,714 bp in total length and is composed of large single copy region (LSC) of 85,402 bp, small single copy region (SSC) of 18,750 bp and two inverted repeat regions (IRa and IRb) of 25,781 bp each. The overall GC content of the chloroplast genome was 37.1%: LSC (35.1%), SSC (31.0%) and IRs (42.8%). The chloroplast genome contained 131 genes, including 86 protein-coding, eight rRNA, and 37 tRNA genes. A total of 17 genes were duplicated in the inverted repeat regions including seven tRNA, four rRNA, and six protein-coding genes. Fifteen genes (*ndh*A, *ndh*B, *pet*B, *pet*D, *rpl*2, *rpl*16, *rpo*C1, *rps*12, *rps*16, *trnA-UGC*, *trnG-UCC*, *trnI-GAU*, *trnK-UUU*, *trnL-UAA*, and *trnV-UAC*) contained one intron, while *clp*P and *ycf*3 contained two introns. Interestingly, highly conserved group II intron of the *atp*F gene was lost. The partial (1092 bp) of *ycf*1 was located in IRb/SSC junction region, while the complete *ycf*1 gene was included in the IR at the SSC/IRa junction. The *inf*A gene located in LSC was pseudogene.

To confirm the phylogenetic position of *R*. *crataegifolius* within Rosaceae, 17 representative species of Rosaceae (*Prinsepia utilis* Royle as outgroup) were aligned using MAFFT v.7 (Katoh and Standley 2013) and maximum-likelihood (ML) analysis was conducted based on the concatenated 68 coding genes using IQ-TREE v.1.4.2 (Nguyen et al. 2015). The ML tree identified strongly supported monophyletic *Rubus* (Figure 1) and a sister relationship between *R. crataegifolius* and *R. corchorifolius* L. in subgenus *Idaeobatus* (Focke 1911).

**Figure 1.**
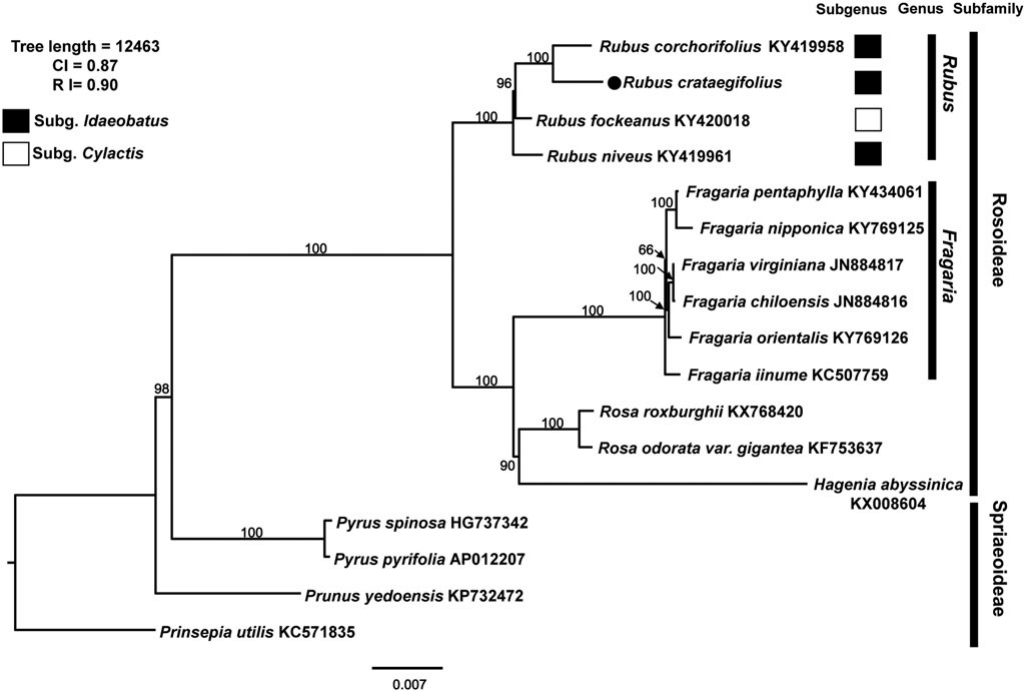
The maximum-likelihood (ML) tree based on 68 protein-coding genes in the 17 representative chloroplast genomes of Rosaceae. The bootstrap value based on 1000 replicates is shown on each node.
